# *LEP* (−2548G>A LEP) and *LEPR* (223Gln>Arg, 109Lys>Arg) polymorphisms as breast cancer risk factors in the Polish female population

**DOI:** 10.1007/s11033-021-06328-7

**Published:** 2021-04-17

**Authors:** Hanna Hołysz, Anna Paszel-Jaworska, Aleksandra Romaniuk-Drapała, Sylwia Grodecka-Gazdecka, Błażej Rubiś

**Affiliations:** 1 Department of Clinical Chemistry and Molecular Diagnostics, Poznan University of Medical Sciences, 49 Przybyszewskiego St., 60-355 Poznan, Poland; 2 Department of Oncology, Poznan University of Medical Sciences, Poznan, Poland

**Keywords:** Leptin, *LEP*, *LEPR*, Single nucleotide polymorphisms, Breast cancer

## Abstract

On a global scale, breast cancer is the most common type of cancer in women, and it is still a growing problem. Therefore, new prognostic or diagnostic markers are required that would facilitate the assessment of patients or provide more efficient therapy, respectively. In these studies, we analyzed the contribution of *LEP* (2548G>A) and *LEPR* (109 Lys>Arg and 223Gln>Arg) genes polymorphisms to the risk of breast cancer development. The study involved 209 women aged 59.6 ± 11 years diagnosed with breast cancer and 202 healthy women aged 57.8 ± 8.2 years, who were blood donors. Polymorphism were evaluated by PCR–RFLP reaction followed by the verification of part of the samples by sequencing. The results of the study confirmed obesity as a significant breast cancer development risk factor in Polish women. However, no significant association between the studied polymorphisms and breast cancer risk or severity of the neoplastic disease was found. Interestingly, it was shown that wild type 223Gln>Gln leptin receptor (LEPR) was statistically more common in women with human epidermal growth factor receptor 2 negative (HER2−) than human epidermal groth factor receptor 2 positive (HER2+) breast cancer and wild type form of 2548G>A LEP was more common in women with progesterone receptor positive (PR+) than progesterone receptor negative (PR−) breast cancer. Studied polymorphisms of the *LEP* and *LEPR* genes do not increase breast cancer risk in the population of Polish women. However, they can affect PR an HER receptors expression and thus the severity of the disease. Noteworthy, this interesting correlation is being reported for the first time and might constitute an essential contribution to the identification of molecular mechanisms of carcinogenesis.

## Introduction

### Leptin

The increasing incidence of breast cancer in both women and men is significantly correlated with obesity [1]. Adipose tissue is the largest organ of endocrine secretion, and it secretes several hormones, including leptin—product of the *LEP* gene [2]. Biological effect of leptin is observed after the association with the leptin receptor, which belongs to the class I of the cytokine superfamily, and is encoded by the *LEPR* gene [3]. The most important function of the leptin-leptin receptor complex is the regulation of hypothalamic centers of hunger reflected in the regulation of energy expenditure and food intake [4]. Leptin has a similar structure to interleukins IL-6, IL-12, IL-15, prolactin, growth hormone, granulocyte colony-stimulating factor (GCSF) and oncostatin M [5]. It is able to regulate the immune system by increasing the proliferation of leukocytes and elevating the production of proinflammatory cytokines [6, 7].

### Leptin in cancer

Interestingly, a positive correlation was found between the leptin level and the incidence of breast cancer, which probably results from the proinflammatory, pro-angiogenic, and pro-carcinogenic activity of this protein. Both in vitro and in vivo studies have shown that leptin stimulates tumor growth and cell migration, thereby increasing the risk of distant metastasis [8, 9]. Leptin may also contribute to the pathway controlled by estrogen receptor alpha in breast cells leading to the induction of aromatase, followed by aromatization of androgens to estrogens. Consequently, increased estrogen levels, as well as increased risk of estrogen-dependent breast cancer, are observed. In addition, leptin prevents the degradation of ER-α (Estrogen Receptor alpha), thereby enhancing its performance [10, 11]. Moreover, it leads to the feedback in which 17beta-estradiol enhances the synthesis of leptin in adipocytes, which, after stimulation of the leptin receptor, increases expression of epidermal growth factor receptor 2 gene (*ERBB2*) [12]. As a result, increased HER2 (Human Epidermal Growth Factor Receptor 2) significantly contributes to the transformation of the gland and correlates with a more aggressive form of the disease and resistance to hormonal therapy. Leptin can also act as a pro-angiogenic and proinflammatory agent, which increases the initiation and progression of cancer. It activates the STAT3 kinase-dependent signaling pathway Janus JAK2/STAT3 (Janus kinase/signal transducers and activators of transcription) leading to increased production of angiogenic factors such as VEGF (Vascular Endothelial Growth Factor) and FGF (Fibroblast Growth Factor), thereby increases vascular endothelial cell proliferation [13]—to teraz cytowanie nr 13. Synergistically with IL-1, it activates NFκB (Nuclear Factor kappa-light-chain-enhancer of activated B cells), provokes an increase in VEGF and enhances angiogenesis, which is one of the most critical factors in tumor development [14]. Leptin stimulates the macrophage secretion of proinflammatory cytokines: IL-1, TNF-alfa (Tumor Necrosis Factor-alfa), IL-12 [6]. An increase of TNF-alfa leads to the inhibition of breast cancer cell proliferation and a reduction of the risk of metastasis. On the other hand, IL-1 stimulates cell division, promoting angiogenesis, tumor growth, and metastasis. In addition, increased leptin and IL-6 were observed in the blood serum of women with breast cancer. Interestingly, the pro-carcinogenic effect of leptin is enhanced by its autocrine actions in tumor cells. It was also shown that the leptin receptor was expressed in breast cancer cells predominantly, relative to normal cells, which correlates with larger tumor size, lymph node and distant metastases, a shorter time free from recurrence of the disease, and a greater degree of disease severity.

### LEP, LEPR, and genetic variants

The increase in serum leptin concentration may be due to excess body fat or a decreased leptin receptor functioning/sensitivity. The production of leptin may also depend on the expression of the *LEP* and *LEPR* genes, which can be affected by polymorphisms [15].

The most significant effect on the function of these proteins seems to be correlated with the polymorphisms in the position 2548G>A of the *LEP* gene (2548G>A, rs7799039) and polymorphisms of the *LEPR* gene in position 326 (326A>G) which results in the conversion of lysine to arginine at position 109 of the leptin receptor (109 Lys>Arg, rs1137100) and position 668 (668A>G) resulting in the conversion of glutamine to arginine at position 223 (223Gln>Arg, rs1137101), respectively [16].

Noteworthy, the frequency of the *LEP* polymorphic allele A (2548G>A) varies in different populations. This polymorphism is found in the gene promoter region, thus can regulate the binding of transcription factors and, consequently, contribute to the change in *LEP* gene expression and serum leptin levels [17]. The presence of AA polymorphic homozygotes and GA heterozygotes may correlate with higher levels of leptin and obesity, and thus with a higher risk of breast cancer [18]. The Lys109Arg and Gln223Arg polymorphisms are located in exons 4 and 6 of the *LEPR* gene encoding the leptin receptor extracellular domain. Replacing amino acids in this part of the protein may interfere with the binding of leptin, which can lead to the lack of a biological effect and increased leptin concentration. The frequency of both polymorphic forms varies between populations [19–21]. In summary, we decided to evaluate the impact of *LEP* and *LEPR* genes polymorphisms on the development of breast cancer in women from the Greater Poland region. Unfortunately, since these were just retrospective studies based on the assessment of already collected blood samples (with no plasma fraction), we could not evaluate the correlation between the leptin level and the *LEP*/*LEPR* genotypes.

## Materials and methods

### Individuals

Based on the population data on the incidence of breast cancer among women from the Greater Poland region the sample size and power was calculated with calculator.net (α = 0.95).

The control group consisted of 202 women in menopausal age (age 57.8 ± 8.2) who were blood donors at The Regional Center for Blood Donation and Blood Treatment in Poznań, Poland. Women were diagnosed as cancer-free.

The health status of blood donors was assessed using: medical history including the age of the donor, past diseases, lifestyle, medicines and treatments; physical examination including body height and weight, body temperature measurement, blood pressure, and heart rate, and assessment of lymph nodes; laboratory blood tests including blood counts, total protein level, presence of HBs antigen, anti-HIV antibodies, anti-HCV antibodies, HCV RNA, HBV DNA, and HIV RNA.

The study group consisted of 209 women aged 59.6 ± 11 hospitalized in the Department of Oncology, Poznan University of Medical Sciences. They were diagnosed with various types of breast cancer at various stages. The most common type of tumor (50.5%) was invasive ductal carcinoma (IDC).

DNA was isolated from blood remaining after standard laboratory test. All women agreed to use their blood for research. The study obtained the permission of the Bioethics Committee of the Poznan University of Medical University, No. 568/18.

### DNA isolation and sequence assessment

**Fig. 1:**
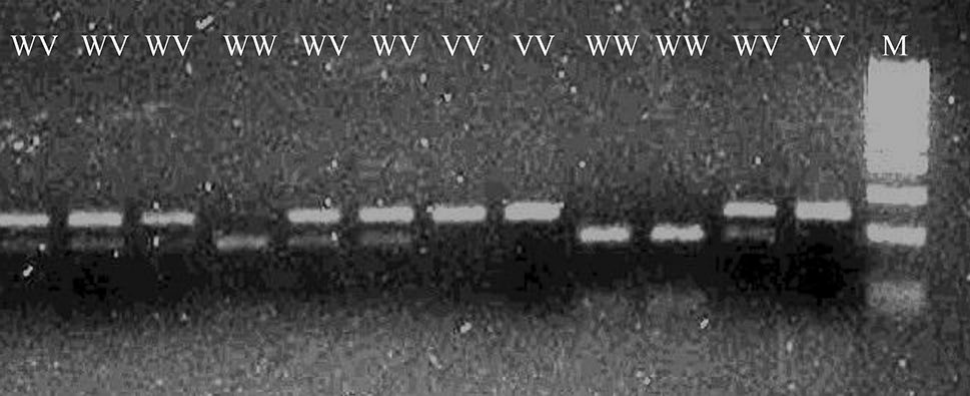
Typical electrophoretic separation of amplicons digested with the restriction enzyme CfoI. The CfoI enzyme recognizes the wild-type GG sequence of the 2548 G>A *LEP* gene polymorphism. *WW* wild type homozygous, *WV* heterozygous, VV polymorphic homozygous, *M* molecular – weight size marker

**Fig. 2:**
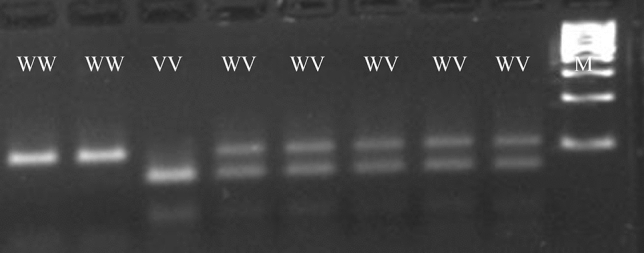
Typical electrophoretic separation of amplicons digested with the restriction enzyme HaeIII. The Hae III enzyme recognizes the polymorphic sequence of the 109Lys>Arg *LEPR* gene polymorphism. *WW* wild type homozygous, *WV* heterozygous, VV polymorphic homozygous, *M* molecular – weight size marker

**Fig. 3:**
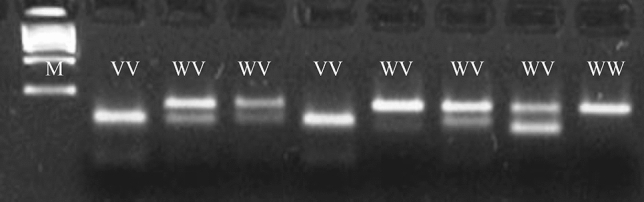
Typical electrophoretic separation of amplicons digested with the restriction enzyme MspI. The MspI enzyme recognizes the polymorphic sequence of the 223 Gln>Arg *LEPR* gene polymorphism. *WW* wild type homozygous, *WV* heterozygous, VV polymorphic homozygous, *M* molecular – weight size marker

DNA was isolated from peripheral blood leukocytes with an isolation kit (A&A Biotechnology, Gdynia, Poland). Amplification of *LEP* and *LEPR* genes fragments was performed using specific primers at the optimal annealing temperature and reaction conditions that were determined experimentally (Table 1). The components of the reaction were mixed and added to a sterile tube to a final volume of 24 µl (Table 2). Then 1 µl of DNA was added and the samples were placed in a thermocycler. The amplification products of *LEP* gene polymorphism with a length 242 bp were digested with a specific restriction enzyme (CfoI) (Fig. 1). Polymorphisms of the *LEPR* gene were also amplified using specific primers in optimal conditions (Tables 1and 2) and the obtained products had length 101 bp (Lys109Arg) and 80 bp (Gln223Arg). Amplification products were digested with specific restriction enzymes HaeIII (Lys109Arg) (Fig. 2) or MspI (Gln223Arg) (Fig. 3). All primers used for the PCR reaction were designed using the program Primer 3 [22]. Both amplification and restriction digest products were separated by agarose electrophoresis and labeled with Midori green.

**Table 1:** Primers sequence, annealing temperature, products length and the percentage of the gel necessary to analyze polymorphisms of the *LEP* and *LEPR* genes

Primers	Primers sequence	Primer binding temperature [^o^C]	Amplicon length [bp]	Agarose gel [%]
LEP F LEP R	5′-TTTCTGTAATTTTCCCGTGAG-3′ 5′-AAAGCAAAGACAGGCATAAAAA-3′	53	242	1,5
LEPR 109Lys>Arg F LEPR 109Lys>Arg R	5′-TTTCCACTGTTGCTTTCGGA-3′ 5′-AAACTAAAGAATTTACTGTTTGAAACAAATGGC-3′	55	101	3
LEPR 223Gln>Arg F LEPR 223Gln>Arg R	5′-AAACTCAACGACACTCTCCTT-3′ 5′-TGAACTGACATTAGAGGTGAC-3′	59	80	3

*F* forward primer, *R* reverse primer, *bp* base pair

**Table 2:** The PCR reaction mixture

Ingridients	Concentration/quantity	Volume [µl]
DNA	50 ng	1
Taq polymerase	5 U/µl	0,2
dNTPs	25 mM	1
F primer	25 μM	0,5
R primer	25 μM	0,5
Buffer Mg^2+^	25 mM	2,5
Water	–	19,3
Final volume		25

*F* forward primer, *R* reverse primer, *dNTPs* deoxynucleotide triphosphates

To assess the specificity of restriction enzymes digestion, 10% of the samples were subjected to sequencing using the BigDye v3.1 kit (Applied Biosys-tems, Darmstadt, Germany) and separation by ethanol extraction using the ABI Prism 3130XL (Applied Biosystems, Darmstadt, Germany) in a 36 cm capillary in a POP7 polymer.

### Statistical analysis

We used Fisher’s exact test to examine the distinction in the genotypic and allelic frequencies between the cases and control. The continuous variables between the studied groups were com-pared with the Student’s *t*-test. The occurrence of genotypes in the study group with different tumor stage and grade was assessed using the Hardy–Weinberg equilibrium (χ^2^ test). Odds ratio (OR) and 95% confidence intervals (CIs) was also calculated. A p-value of < 0.05 was considered statistically significant (Statistica 6.0).

## Results

### Analysis of the frequency of polymorphisms

The observed genotype frequency distribution did not show significant deviation from Hardy Weinberg equilibrium (Table 3). The analysis of the bodyweight of the study and control subjects showed that women suffering from breast cancer had a statistically higher body mass index (BMI) than healthy women, i.e., 26.6 ± 4.29 vs. 24.94 ± 4.18 (p = 0.001) (Table 4). These studies confirm that obesity is an important factor contributing to the development of breast cancer, also in women from Poland. Expression of estrogen and progesterone receptors has been demonstrated in 81% (ER+) and 73% (PR+) breast cancer tissues of women, and these values were higher than indicated in other populations [23]. The research also showed that most of the patients were diagnosed at an early stage i.e., primary tumor size in 71% of women was smaller than 2 cm, and 54% of women did not show any lymph node metastases (Table 5).

**Table 3:** Genotypes frequencies and results of comparison with Hardy–Weinberg expectations

Gene SNP	Genotype	Patients (n = 209)			Controls (n = 202)		
		Genotypic frequency	Expected frequency	χ^2^	Genotypic frequency	Expected frequency	χ^2^
		%	%		%	%	
*LEP* rs7799039 2548 G>A	GG	33	30	1,758	34	33	0,167
	GA	43,5	50		47	49	
	AA	23,5	20		19	18	
*LEPR* rs1137101 223Gln>Arg	Gln,Gln	31,5	33	0,575	30	29	0,199
	Gln,Arg	52,5	49		47,5	50	
	Arg,Arg	16	18		22,5	21	
*LEPR* rs1137100 109Lys>Arg	Lys,Lys	47	47,5	0,08	50,5	47,5	1,825
	Lys,Arg	44	43		37	43	
	Arg,Arg	9	9,5		12,5	9,5	

Results statistically significant are those for which the level of significance was less than 0.05

**Table 4:** The studied and control group characteristics

	Patients (n = 209)	Controls (n = 202)	p
Age	59.6 ± 11	57.8 ± 8.2	** < 0.001**
Weight (kg)	70.5 ± 12.1	66.5 ± 11.7	** < 0.001**
Height (cm)	162.6 ± 6.1	136.3 ± 5.4	0.201
BMI	26.6 ± 4.29	24.94 ± 4.18	** < 0.001**
BMI < 18,49	0	0	-
BMI 18,5–24,99	39% (83)	55% (112)	** < 0.001**
BMI > 25	61% (126)	45% (90)	** < 0.001**

BMI < 18,49 – underweight, BMI 18,5–24,99 correct body weight, BMI > 25 overweight; Based on the World Obesity Federation. Bold values denote statistical significance at the p < 0.05 level

*BMI* Body Mass Index

**Table 5:** Histological and clinical characteristics of breast tumors

Parameter	Symbol	% (n)
Estrogen receptor	ER+	81 (169)
	ER−	19 (40)
Progesterone receptor	PR+	73 (153)
	PR−	27 (56)
Human epidermal growth factor receptor 2	HER+	18 (38)
	HER−	82 (171)
Histological tumor grade	G1	35 (73)
	G2	40 (84)
	G3	20 (42)
	Gx	5 (10)
Tumor size	Tis	2 (3)
	T1	71 (148)
	T2	21 (43)
	T3	0 (0)
	T4	2 (4)
	Tx	5 (10)
Nodules	N0	54 (113)
	N1	35(74)
	N2	4 (8)
	N3	2 (4)
	Nx	5 (10)

*ER* estrogen receptor, *PgR* progesterone receptor, *HER2* human epidermal growth factor Receptor 2, *G* histological grade, *T* tumor size, *N* lymph node metastasis

Analysis of the results did not show a statistically significant difference between the frequency of polymorphisms of the *LEP* (2548G>A) and the *LEPR* gene (109Lys>Arg) in the group of women with breast cancer and healthy women. Genotype distributions and allele frequencies of the leptin gene 2548G>A polymorphism in the group of women with breast cancer were 34% for WW, 47% for WV, 19% for VV, and in the group of healthy women it was 33% 43.5%, and 23.5% respectively.

Individual genotypes of *LEPR* polymorphism 109 Lys>Arg occur in the group of women with breast cancer with frequency 47% for WW, 44% for WV, 9%for VV, and the group of control subjects 50.5%, 37%, and 12.5%. These differences are also not statistically significant. However, analysis of polymorphism 223Gln>Arg showed that the polymorphic allele occurred statistically more often in the control group than in the study group (p = 0.05) (Table 6).

**Table 6:** Incidence of *LEP* and *LEPR* genes polymorphisms in healthy women and breast cancer patients

Gen SNP	Genotype	Patients		Controls		WW vs. WV, VV	WW, WV vs. VV	W vs. V
		n = 209	%	n = 202	%			
*LEP* rs7799039 2548 G>A	GG	71	34	67	33	p = 0.9153	p = 0.2162	p = 0.3909
	GA	98	47	88	43,5			
	AA	40	19	47	23,5			
*LEPR* rs1137101 223Gln>Arg	Gln,Gln	66	31,5	61	30	p = 0.8280	**p = 0.0500**	p = 0.2233
	Gln,Arg	110	52,5	96	47,5			
	Arg,Arg	33	16	45	22,5			
*LEPR* rs1137100 109Lys>Arg	Lys,Lys	98	47	102	50.5	p = 0.6168	p = 0.0935	p = 0.6997
	Lys,Arg	92	44	75	37			
	Arg,Arg	19	9	25	12,5			

Bold values denote statistical significance at the p < 0.05 level

*WW* wild type homozygous, *WV* heterozygous, *VV* polymorphic homozygous

### Association between polymorphisms and body mass index

Leptin is produced by adipose tissue which amount directly affects plasma concentration of the protein. The influence of *LEP* and *LEPR* genes polymorphisms on the body mass of women with breast cancer and control subjects was assessed. It was shown that the polymorphic allele 109Arg>Arg (*LEPR*) correlated with the increased body weight of healthy women (WW, WV vs. VV p = 0.0599). However, there was no association between *LEP* (2548G>A) and *LEPR* (223Gln>Arg) genes polymorphisms and the body mass index weight in both groups (data not shown).

### Association between polymorphisms and breast cancer advancement

The influence of the examined genes polymorphisms on the characteristics of tumor cells, breast cancer advancement, and tumor grade was also assessed. The cases were characterized for the presence of receptors ER, PR, and HER2, and the stage of breast cancer was assessed based on the size of the primary tumor and lymph nodes metastasis. Breast cancer cells in patients who were characterized as wild type homozygotes 223Gln>Gln (*LEPR*) were shown to significantly less frequently express HER2 (HER2+ 24.5% vs. HER2− 46.5%). Moreover, patients who were wild type homozygotes 2548 G>G (*LEP*) were statistically more likely to express PR receptors than women who were heterozygous and polymorphic homozygous. However, no association between the polymorphism and ER or PR expression was observed. There was also no relationship between *LEPR* 109Lys>Arg polymorphisms and the expression of ER, PR, and HER2 receptors (Table 7). Moreover, statistical analysis did not show differences in the cancer stage and grade in patients with different genotypes (data not shown).

**Table 7:** Relationship between *LEP* and *LEPR* gene polymorphisms and expression of estrogen (ER), progesterone (PR) and epidermal growth factor (HER2) receptors

Gen SNP	Genotype	ER+ (%)	ER− (%)	WW vs. WV, VV	WW, WV vs. VV	W vs. V	PR+ (%)	PR− (%)	WW vs. WV, VV	WW, WV vs. VV	W vs. V	HER+ (%)	HER− (%)	WW vs. WV, VV	WW, WV vs. VV	W vs. V
*LEP* rs7799039 2548 G>A	GG	38	17	p = 0.0591	p = 1.000	p = 0.2619	39.5	17.5	**p = 0.0227**	p = 0.6206	p = 0.2601	26	30	p = 0.8345	p = 0.6330	p = 0.5938
	GA	44	67				41	67.5				53	53			
	AA	18	17				19.5	15				21	17			
*LEPR* rs1137101 223Gln>Arg	Gln,Gln	35	42	p = 0.6431	p = 0.3511	p = 1.000	35	41	p = 0.5435	p = 0.5846	p = 0.8878	24.5	46.5	**p = 0.0324**	p = 0.1634	p = 0.0199
	Gln,Arg	51	37.5				51	41				51	45			
	Arg,Arg	14	20.5				14	18				24.5	8.5			
*LEPR* rs1137100 109Lys>Arg	Lys,Lys	44.5	50	p = 0.6584	p = 0.6908	p = 0.6090	42	56	p = 0.1734	p = 0.4597	p = 0.1353	45	43	p = 0.8517	p = 1.000	p = 1.000
	Lys,Arg	47	45				49	41				45	48			
	Arg,Arg	8.5	5				9	3				10	9			

Bold values denote statistical significance at the p < 0.05 level

*ER* estrogen receptor, *PgR* progesterone receptor, *HER2* human epidermal growth factor receptor 2, *WW* wild type homozygous, *WV* heterozygous, VV polymorphic homozygous

## Discussion

Breast cancer is the most commonly diagnosed cancer in women and is the second cause of their death. One of the most important risk factors is obesity [24], probably due to the fact that adipose tissue produces many factors/proteins that can initiate carcinogenesis. Moreover, studies show that adipose tissue can influence tumor growth and metastasis. One of many proteins produced by adipose tissue is leptin, a cytokine that has been characterized as a growth factor in breast cancer. Leptin levels depend on the amount of adipose tissue but also on the expression of the *LEP* and *LEPR* genes [25]. We showed that BMI higher than 25 was found in 45% of Polish women from the Greater Poland region. This result corresponds to the nationwide data indicating overweight and obesity in 46% of the Polish population. However, these data are lower than global reports since WHO estimates that 52% of the world's adult population was overweight and obese in 2016 [26]. Importantly, most of those cases are women. It is known that every reduction of 5 kg of body weight decreases the risk of breast cancer by 8%.

It is possible that lower plasma concentration of the hormone, resulting from the lower amount of adipose tissue, reduces the risk of breast cancer development. However, some women show high levels of leptin independent of the amount of adipose tissue, which may result from *LEP* and *LEPR* gene polymorphisms [27]. Women with average body weight who are 2548AA polymorphic homozygotes have higher blood leptin levels than wild 2548GG homozygotes and 2548GA heterozygotes [18]. However, in obese women (Tunisian population) this relationship was reversed. Lower leptin levels were observed in subjects carrying the 2548A allele than in those with GG genotype and this relation was not noticed in the control, normal weight, group [17]. Therefore, it seems that leptin level may depend not only on *LEP* and *LEPR* polymorphisms but also on environmental factors. As already mentioned, this study was performed based on the assessment of already collected blood samples (with no plasma fraction). Additionally, women with different body weight and BMI were enrolled in the study. Consequently, we definitely lack a broader picture of the potential correlation between genetic and environmental factor which implies further detailed studies in the context of breast cancer risk.

Importantly, no differences were found in the incidence of 2548G>A polymorphism in healthy women with average and high body mass index (BMI>25) (WW vs. WV + VV OR = 1.087, p = 0.8784; WW + WV vs. VV, p = 0.3956), and the results are comparable to the Mexican and Romanian populations [28, 29].

Furthermore, there were no statistically significant differences in the incidence of individual polymorphisms 223Gln>Arg and 109Lys>Arg of the *LEPR* gene among healthy women with normal and high (BMI > 25) body weight. The influence of 223Gln>Arg polymorphism on the body mass index of women with breast cancer also has not been demonstrated. However, it was shown that 109Arg>Arg polymorphisms occur significantly more often in individuals with breast cancer and normal weight than in overweight and obese women. Therefore, it is possible, that the presence of arginine in the leptin receptor results in a change of the structure of the leptin receptor and thus the binding of leptin [30]. Eventually, it may lead to resistance to leptin and increased plasma concentration of this hormone, which may initiate the neoplastic process. Our studies did not show any statistically significant differences between 2548G>A polymorphism in the group of control women and breast cancer patients. However, it was shown that the presence of the 223Arg>Arg polymorphic allele might correlate with a lower risk of breast cancer. Healthy women significantly more frequently have polymorphic allele 223Arg>Arg than breast cancer patients. On the other hand, there were no differences in the frequency of the wild 223Gln allele in the population of healthy controls and patients. However, studies conducted by El-Hussina et al. and Han et al. showed that the wild-type Gln allele correlated with a higher risk of breast cancer development [31, 32]. Wild homozygotes 223Gln>Gln are statistically more frequent in the group of female patients than in the group of healthy Egyptians (18.8% vs. 4.2%) [31] as well as Chinese (13.7% vs. 2.3%) [32]. It may result from differences in the population distribution of the wild type allele, which in the Polish population is found in 30% women. Among Egyptians and Chinese, it is 4.2% and 2.37%, respectively [31, 32].

It is suggested that leptin may induce estrogen receptor expression directly and indirectly by increasing estrogen levels. Moreover, estrogen modulates leptin receptor expression and, as a result, affects leptin levels [33]. In vitro studies indicate an increased cell proliferation in leptin-induced MCF7 (ER+, PR+, HER2−). Therefore, the relationship between *LEP* and *LEPR* gene polymorphisms and expression of ER, PR, and HER2 receptors in women with breast cancer was evaluated. It was shown, that wild type 223Gln>Gln *LEPR* is statistically more common in women with HER2 negative breast cancer (W vs. V p = 0.019) and wild type form of 2548G>A LEP is more common in women with PR positive breast cancer (WW vs. WV, VV p = 0.022). (Table 7) None of the available literature position indicates such a relationship. Mahmouidi et al. assessed the relationship between 223Gln>Arg *LEPR* and ER, PR, and HER2 expression in the Egyptian population and showed no significant differences [34]. These differences may, however, depend on the studied population. Analysis of the influence of *LEP* and *LEPR* genes polymorphisms on the stage of breast cancer did not show any relationship between polymorphisms and the size of the tumor, lymph node metastases, or tumor grade.

## Conclusions

Statistical analysis confirmed that obesity is a significant risk factor for breast cancer in Polish menopausal women from The Grater Poland region. This relationship positively correlates with data from other populations. 109Lys>Arg *LEPR* gene polymorphism correlates with obesity in healthy women but does not increase the risk and stage of breast cancer. Moreover, the occurrence of *LEPR* gene polymorphisms (*LEPR* Gln>Arg) correlated with HER2 receptor expression in breast cancer cells of women from Greater Poland. However, the conducted analyzes did not show the relationship between the *LEP* and *LEPR* gene polymorphisms and the risk of breast cancer development and its stage. To the best of our knowledge, this is the first study to evaluate the correlation between LEP/LEPR polymorphisms status and breast cancer in the population of women from Greater Poland.

## References

[1] Basen-Engquist K, Chang M (2011). Obesity and cancer risk: recent review and evidence. Curr Oncol Rep.

[2] Nalabolu MR, Palasamudram K, Jamil K (2014). Adiponectin and leptin molecular actions and clinical significance in breast cancer. Int J Hematol Stem Cell Res.

[3] Bjørbaek C (2009). Central leptin receptor action and resistance in obesity. J Investig Med.

[4] Albuquerque D, Stice E, Rodríguez-López R (2015). Current review of genetics of human obesity: from molecular mechanisms to an evolutionary perspective. Mol Genet Genomics.

[5] Giordano C, Barone I, Bonofiglio D (2019). Obesity, leptin and breast cancer: epidemiological evidence and proposed mechanisms. Cancers (Basel).

[6] Fernández-Riejos P, Najib S, Santos-Alvarez J (2010). Role of leptin in the activation of immune cells. Mediat Inflamm.

[7] Faggioni R, Feingold KR, Grunfeld C (2001). Leptin regulation of the immune response and the immunodeficiency of malnutrition. FASEB J.

[8] Gonzalez-Perez RR, Lanier V, Newman G (2013). Leptin’s pro-angiogenic signature in breast cancer. Cancers (Basel).

[9] Gyamfi J, Eom M, Koo JS, Choi J (2018). Multifaceted roles of interleukin-6 in adipocyte–breast cancer cell interaction. Transl Oncol.

[10] Lee K-M, Noh D-Y, Yom CK (2013). Leptin as a potential target for estrogen receptor-positive breast cancer. J Breast Cancer.

[11] Surmacz E (2007). Obesity hormone leptin: a new target in breast cancer?. Breast Cancer Res.

[12] Cha Y, Kang Y, Moon A (2012). HER2 induces expression of leptin in human breast epithelial cells. BMB Rep.

[13] Ohba S, Lanigan TM, Roessler BJ (2010). Leptin receptor JAK2/STAT3 signaling modulates expression of Frizzled receptors in articular chondrocytes. Osteoarthr Cartil.

[14] Lipsey CC, Harbuzariu A, Daley-Brown D, Gonzalez-Perez RR (2016). Oncogenic role of leptin and Notch interleukin-1 leptin crosstalk outcome in cancer. World J Methodol.

[15] Tomas Ž, Petranović MZ, Škarić-Jurić T (2014). Novel locus for fibrinogen in 3′ region of LEPR gene in island population of Vis (Croatia). J Hum Genet.

[16] Knuła H, Rubiś B, Rybczyńska M (2009). The roles of leptin and LEP and LEPR gene polymorphisms in pathogenesis of breast cancer. Wspolczesna Onkol.

[17] Ben Ali S, Kallel A, Ftouhi B (2009). Association of G-2548A LEP polymorphism with plasma leptin levels in Tunisian obese patients. Clin Biochem.

[18] Hoffsted J, Eriksson P, Mottagui-Tabar S, Arner P (2002). A polymorphism in the leptin promoter region (-2548 G/A) influences gene expression and adipose tissue secretion of leptin. Horm Metab Res.

[19] Lu J, Zou D, Zheng L (2013). Synergistic effect of LEP and LEPR gene polymorphism on body mass index in a Chinese population. Obes Res Clin Pract.

[20] Ma González Huerta L, Santos Cabrera CI, Mociños Montes R (2017). Association between leptin and leptin receptor gene polymorphisms and breast cancer risk in premenopausal and postmenopausal Mexican women. Cancer Res Front.

[21] Brandl EJ, Frydrychowicz C, Tiwari AK (2012). Association study of polymorphisms in leptin and leptin receptor genes with antipsychotic-induced body weight gain. Prog Neuro-Psychopharmacol Biol Psychiatry.

[22] Primer3 Input (version 0.4.0). http://bioinfo.ut.ee/primer3-0.4.0/

[23] Warner ET, Tamimi RM, Hughes ME (2012). Time to diagnosis and breast cancer stage by race/ethnicity. Breast Cancer Res Treat.

[24] Protani M, Coory M, Martin JH (2010). Effect of obesity on survival of women with breast cancer: systematic review and meta-analysis. Breast Cancer Res Treat.

[25] Lorincz AM, Sukumar S (2006). Molecular links between obesity and breast cancer. Endocr Relat Cancer.

[26] Ritchie H, Roser M (2017) Obesity. https://ourworldindata.org/obesity

[27] Wauters M, Considine RV, Chagnon M (2002). Leptin levels, leptin receptor gene polymorphisms, and energy metabolism in women. Obes Res.

[28] Chavarria-Avila E, Vázquez-Del Mercado M, Gomez-Bañuelos E (2015). The impact of LEP G-2548A and LEPR Gln223Arg polymorphisms on adiposity, leptin, and leptin-receptor serum levels in a Mexican Mestizo population. Biomed Res Int.

[29] Constantin A, Glavce CS, Vladica M (2010). Leptin G-2548A and leptin receptor Q223R gene polymorphisms are not associated with obesity in Romanian subjects. Biochem Biophys Res Commun.

[30] Carrillo-Vázquez JP, Chimal-Vega B, Zamora-López B (2013). Structural consequences of the polymorphism Q223R in the human leptin receptor: a molecular dynamics study. Am J Agric Biol Sci.

[31] El-Hussiny MAB, Atwa MA, Rashad WE (2017). Leptin receptor Q223R polymorphism in Egyptian female patients with breast cancer. Wspolczesna Onkol.

[32] Han CZ, Du LL, Jing JX (2008). Associations among lipids, leptin, and leptin receptor gene gin223arg polymorphisms and breast cancer in China. Biol Trace Elem Res.

[33] Jenks MZ, Fairfield HE, Johnson EC (2017). Sex steroid hormones regulate leptin transcript accumulation and protein secretion in 3T3-L1 cells. Sci Rep.

[34] Mahmoudi R, Noori Alavicheh B, Nazer Mozaffari MA (2015). Polymorphisms of leptin (-2548 G/A) and leptin receptor (Q223R) genes in Iranian women with breast cancer. Int J Genomics.

